# Tisotumab vedotin extravasation injury in a patient with recurrent cervical cancer

**DOI:** 10.1016/j.gore.2024.101525

**Published:** 2024-10-02

**Authors:** Ji Son, Katherine E. Cain, Claire A. Marten, Kaitlin W Dwyer, Travis T. Sims, Jolyn S. Taylor

**Affiliations:** a Department of Gynecologic Oncology and Reproductive Medicine, The University of Texas MD Anderson Cancer Center, Houston, TX, USA; b Department of Pharmacy Clinical Programs, The University of Texas MD Anderson Cancer Center, Houston, TX, USA

## Abstract

• Infusion site extravasation injury is a rare but serious adverse event of tisotumab vedotin.
• Central venous access device should be considered in patients with risk factors receiving antibody drug conjugate therapy.
• Topical steroids may be used as part of supportive measures.

## 1 Introduction

Tisotumab vedotin (TV) is an antibody-drug conjugate (ADC) targeting tissue factor. TV is composed of tissue factor-targeted human monoclonal immunoglobulin G1 conjugated via a protease-cleavable linker to the drug monomethyl auristatin E (MMAE), an antimitotic agent that targets tubulin pathway. (Doronina et al., 2003, Hamblett et al., 2004) TV is selectively internalized by human tissue factor-expressing tumor cells where it undergoes lysosomal degradation resulting in release of the cytotoxic payload. TV can also induce bystander killing from diffusion of free MMAE into surrounding tumor cells. (de Bono et al., 2019).

Clinically, TV has shown exceptional activity in recurrent cervical cancer and was granted accelerated approval by the FDA. (Hong et al., 2020, Coleman et al., 2021) In phase 1 and 2 study of 55 and 102 patients, respectively, the objective response rate of TV monotherapy was 22–24 %, (Hong et al., 2020, Coleman et al., 2021) which significantly outperformed the historical benchmark of 5–14 %. (Muggia et al., 2004, Bookman et al., 2000, Miller et al., 2008, Schilder et al., 2005, Look et al., 1996, Garcia et al., 2007) With interim analysis of the confirmatory phase 3 trial showing improved overall survival compared to investigator’s choice chemotherapy, (Vergote et al., 2023) as well as ongoing combination trials in earlier lines of therapy, (Lorusso et al., 2022) TV has become an important therapeutic option in cervical cancer. With increased utilization of TV, however, rare complications are now being detected. We report a grade 3 infusion site extravasation injury of TV in a patient with recurrent cervical cancer managed with conservative measures. We then discuss the extravasation category of novel agents and potential mitigating strategies.

## 2 Case

**Fig. 1:**
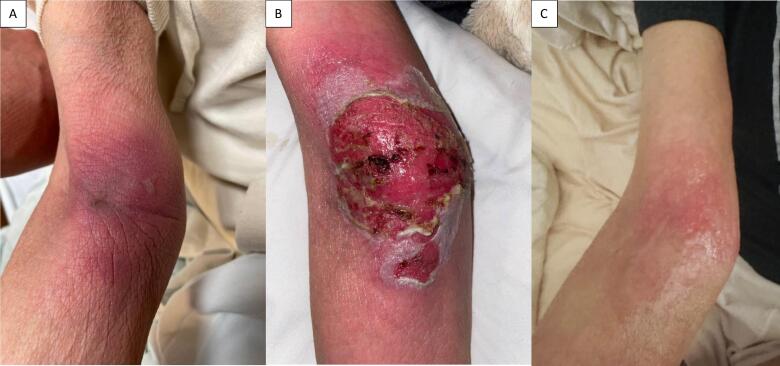
Tisotumab vedotin extravasation injury in a patient with recurrent cervical cancer: (a) 5 days post-infusion, (b) 13 days post-infusion, (c) 11 weeks post-infusion.

A 62 year old woman was initially diagnosed with stage IVB PDL1 + squamous cell carcinoma of the cervix with bone, lung, and liver metastases. She underwent combination therapy with carboplatin, paclitaxel, bevacizumab, and pembrolizumab for ten cycles followed by bevacizumab maintenance and whole brain radiotherapy for brain metastasis. She had progression after four months with new metastatic lesions in multiple lymph nodes, liver, and lung. She was started on TV at 2 mg/kg dose every 21 days. During cycle two of TV, her peripheral intravenous line in the right antecubital vein infiltrated after 23 ml of therapy. Upon recognition, this line was removed, and a new peripheral line was started on the left forearm with infusion of the remainder of the medication. Five days later, the patient was seen in clinic for mild erythema and pain over the infiltration site (Fig. 1). She was started on topical clindamycin and cold compresses. Her symptoms continued, and twelve days post-infusion, she developed pustules and sloughing, which prompted admission to the hospital.

On admission, several services were consulted. Plastic surgery team did not recommend debridement. Given significant pain at the site, dermatology team recommended twice daily application of topical clobetasol 0.05 %. Palliative medicine service optimized pain regimen including opioids. Wound ostomy nursing recommended Mepitel® One Silicone and PolyMem Silver® dressing. She was discharged on hospital day two in improved condition. To avoid further extravasation, patient underwent an implanted central venous access device (CVAD) placement, prior to cycle three of TV. Her positron emission tomography scan after cycle three showed continued response with metabolic resolution of liver metastasis and decrease in the extent of intrathoracic, skeletal, and nodal lesions. By cycle four, seven weeks later, ulceration of the arm was noted to have healed, leaving overlying discoloration and fibrosis.

## 3 Discussion

Extravasated drugs are classified according to their potential for causing damage as irritant, vesicant, or neutral. (Ener et al., 2004) An irritant is a drug that can cause inflammation, pain, or irritation, while a vesicant may cause tissue necrosis or formation of blisters. According to European Society of Medical Oncology guidelines for management of extravasation injury, patient-related risk factors for extravasation in this patient includes likely fragile or sclerosed veins due to multiple therapies in the prior line. Other general risk factors include impaired circulation, coagulopathy, obesity, sensory deficit, decreased ability to communicate, and prolonged infusion time. A suggested preventive measure that applies to this patient is the avoidance of veins in the antecubital fossa or dorsum of the hand, particularly for vesicant drugs. (Pérez Fidalgo et al., 2012) No randomized trials exist on treatment of extravasation injury but guidelines include prompt identification and initiation of general measures such as gentle attempt to aspirate the agent prior to removing the canula. Cold or warm compresses are recommended depending on agent type along with administration of specific resorptive agents or antidotes, if any. In our patient, conservative management with pain control and wound care was successful without surgical intervention. Topical steroid, in particular, seemed to help with pain without significant adverse effects.

Due to the rarity of the complication, extravasation injury in novel agents such as ADCs has not been categorized. Because ADCs are composed of monoclonal antibodies linked to a payload, the cytotoxic potential of both components should be considered. Monoclonal antibodies are generally categorized as neutral, (Pérez Fidalgo et al., 2012) largely based on case reports. For example, nivolumab is considered neutral while bevacizumab has been reported as an irritant. (Rodríguez-Alarcón and Conde-Estévez, 2021) Taxanes, which are similar to the MMAE payload in TV, can have vesicant-like effects depending on concentration and duration. (Barbee et al., 2014) Hence, categorization of ADCs may differ from their monoclonal antibody composition.

There are three case reports on delayed extravasation injury of ADCs, two in trastuzumab-emtansine, a HER2 ADC linked to the cytotoxic DM1 payload. (Sallevelt et al., 2020, Shafaee et al., 2017) Both reports are of heavily pre-treated patients with breast cancer who received peripheral infusion of trastuzumab-emtansine. One event was noted to occur at the antecubital fossa. Both cases progressed over days to weeks, and one of the patients required surgical intervention. There is one case report of extravasation of enfortumab vedotin, which is a nectin 4 ADC using a similar MMAE payload. (Grant et al., 2023) This paper reports on two patients with bladder cancer who experienced extravasation injury. Both patients developed blistering one to three days after extravasation and improved after one to three weeks without special interventions other than general supportive measures. Nectin 4 belongs to a family of cellular adhesion molecules that has been used as an oncogenic target. This molecule is also highly expressed in skin. (Lacouture et al., 2022) Tissue factor, on the other hand, is more selectively expressed in the skin, localized to the epidermis as well vascular adventitia. (Drake et al., 1989) Tissue factor in keratinocytes has been shown to be upregulated in response to injury. (Chen et al., 2005).

While theoretical mechanisms of tissue injury in ADC extravasation have been proposed- for example, proteolysis of ADCs in cutaneous cells or the role of dermal macrophages, (Sallevelt et al., 2020) the exact mechanism is unknown. Once cleaved, TV is designed to induce bystander killing by diffusion, which makes extravasation injury particularly problematic. In innovaTV 204, one patient had a grade 3 extravasation injury, (Coleman et al., 2021) but no further information is available.

While the development and increased utilization of novel therapeutics such as TV is providing unprecedented treatment opportunity for patients, the emerging post-marketing side effects must be closely monitored and managed. We suggest avoidance of antecubital fossa access for infusion of TV in all patients and implanted CVAD in patients with risk factors. Topical steroids may be used as part of supportive measures in extravasation injury. Furthermore, extravasation category of ADCs should consider both the monoclonal antibody and payload components.

Informed Consent Statement.

Informed consent for publication of this report including the deidentified photographs was obtained from the patient and her next-of-kin.

**Author Contribution**

All authors qualified to have authorship are listed in the manuscript in order of contribution. J.S. performed the conceptualization, data curation, formal analysis, and manuscript writing. K.E.C., C.A.M., K.W.D. performed data curation and manuscript editing. T.T.S. performed manuscript editing. J.S.T. performed conceptualization, supervision, formal analysis, and manuscript editing.

## CRediT authorship contribution statement

**Ji Son:** Writing – review & editing, Writing – original draft, Formal analysis, Data curation, Conceptualization. **Katherine E. Cain:** Writing – review & editing, Resources. **Claire A. Marten:** Writing – review & editing, Resources. **Kaitlin W Dwyer:** Writing – review & editing, Resources. **Travis T. Sims:** Writing – review & editing. **Jolyn S. Taylor:** Writing – review & editing, Supervision, Methodology, Data curation, Conceptualization.

## Declaration of competing interest

The authors declare that they have no known competing financial interests or personal relationships that could have appeared to influence the work reported in this paper.
